# The ST2/IL-33 Axis in Immune Cells during Inflammatory Diseases

**DOI:** 10.3389/fimmu.2017.00475

**Published:** 2017-04-24

**Authors:** Brad Griesenauer, Sophie Paczesny

**Affiliations:** ^1^Department of Pediatrics, Indiana University, Indianapolis, IN, USA; ^2^Department of Microbiology Immunology, Indiana University, Indianapolis, IN, USA; ^3^Melvin and Bren Simon Cancer Center, Indiana University, Indianapolis, IN, USA

**Keywords:** IL-33, ST2, IL1RL1, graft-versus-host disease, cardiac diseases, lung diseases

## Abstract

Il1rl1 (also known as ST2) is a member of the IL-1 superfamily, and its only known ligand is IL-33. ST2 exists in two forms as splice variants: a soluble form (sST2), which acts as a decoy receptor, sequesters free IL-33, and does not signal, and a membrane-bound form (ST2), which activates the MyD88/NF-κB signaling pathway to enhance mast cell, Th2, regulatory T cell (Treg), and innate lymphoid cell type 2 functions. sST2 levels are increased in patients with active inflammatory bowel disease, acute cardiac and small bowel transplant allograft rejection, colon and gastric cancers, gut mucosal damage during viral infection, pulmonary disease, heart disease, and graft-versus-host disease. Recently, sST2 has been shown to be secreted by intestinal pro-inflammatory T cells during gut inflammation; on the contrary, protective ST2-expressing Tregs are decreased, implicating that ST2/IL-33 signaling may play an important role in intestinal disease. This review will focus on what is known on its signaling during various inflammatory disease states and highlight potential avenues to intervene in ST2/IL-33 signaling as treatment options.

## Introduction

In 1989, the Il1rl1 gene product, which has also been given the alias ST2 and defined as the IL-33 receptor, as it binds to IL-33, was discovered (1, 2). It belongs to the IL-1-receptor superfamily. The literature has been misnaming ST2 as “suppressor of tumorigenicity 2,” when in fact the original name was “growth stimulation expressed gene 2” (2) and has recently been renamed by the original discoverer, Shin-ichi Tominaga, as “serum stimulation-2” (3), as it was first discovered to function as a mediator of type 2 inflammatory responses (4). IL1RL1 is located on chromosome 2q12.1 in humans, while the gene “suppressor of tumorigenicity 2,” also called ST2, is located on chromosome 11p14.3-p12 in humans. In this review, we will call ST2 as the IL-33 receptor or Ilr1rl gene product.

ST2 has two main splice variants due to differential promoter binding: a membrane-bound form (ST2), which promotes NF-κB signaling, and a soluble form (sST2), which prevents its signaling. It was not until 2005 that the ligand for ST2, the cytokine IL-33, was identified through database searching for genes homologous to other IL-1 superfamily members (5, 6). IL-33 has been identified as a mediator of various inflammatory diseases such as asthma, cardiovascular diseases, and allergic diseases (6). Besides being secreted, IL-33 can be found in the nucleus of human high endothelial venules (7), lung airway epithelium, keratinocytes, fibroblastic reticular cells, and some epithelial cells of the stomach and salivary glands (8). Due to the presence of a *N*-terminal domain nuclear localization sequence and a homeodomain-like helix-turn-helix motif, IL-33 is able to bind heterochromatin, potentially giving IL-33 transcriptional regulatory capacity (7).

Dysregulation of ST2/IL-33 signaling and sST2 production have been implicated in a variety of inflammatory diseases such as cardiac disease (9–12), inflammatory bowel disease (IBD) (13–16), graft-versus-host disease (GVHD) (17–24), small bowel transplant rejection (25), and type 2 diabetes (26–29). The purpose of this review is to highlight the function of both ST2 and sST2, ST2/IL-33 in regard to different immune cells, and sST2 production and ST2 signaling in inflammatory diseases.

## Two Main Isoforms of ST2: Secreted and Membrane Bound on Immune Cells with Opposite Roles

The ST2 gene is located on human chromosome 2q12.1 and is approximately 40 kb long. Homologs of ST2 are found in the genomes of mouse, rat, and fruit fly. ST2 has four splice isoforms from a single transcript dependent on the promoter being used: ST2, a membrane receptor; sST2, a soluble factor; ST2V, a variant form of ST2; and ST2LV, another variant form of ST2, which are differentially regulated through alternative promoter binding (30–32). Little is known about ST2V other than it is expressed highly in gastrointestinal organs (33). ST2LV lacks the transmembrane domain found in ST2; is secreted by eye, heart, lung, and liver tissues; and is found during later stages of embryogenesis (34). Other information on ST2LV is currently lacking.

**Figure 1:**
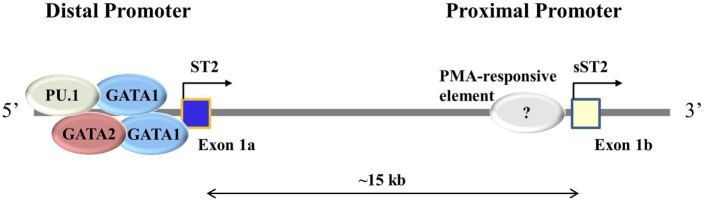
**Different promoter usage dictates ST2 and sST2 expressions**. ST2 consists of two main splice isoforms: ST2 and sST2. These isoforms are splice variants of each other regulated by alternative promoter bindings, the distal promoter for ST2, and the proximal promoter for sST2. Exon 1 varies between ST2 and sST2 depending on the promoter being bound. In immune cells, GATA1, GATA2, and PU.1 have been shown to bind to the distal promoter. The proximal promoter has not been well studied; it is thought that a PMA-responsive element induced sST2 transcription (37).

By cloning the *Il1rl1* gene in rat and sequencing sST2 and ST2 cDNAs, it was found that sST2 and ST2 have different exon 1 sequences (30). Mapping the promoter regions for *Il1rl1* showed that the transcription start site for sST2 is in a proximal promoter region while the transcription start site for ST2 is in a distal promoter region, 15 kb upstream from the sST2 proximal promoter (30) (Figure 1). Three to four GATA transcription factors have been identified at the distal promoter region within 1,001 bp, two of which were conserved between human and mouse *Il1rl1* genes (32, 35). These GATA elements binding to the distal promoter lead to ST2 expression. The transcription factor PU.1 also binds to the distal promoter near the GATA elements in both human mast cells and basophils (36). PU.1 and GATA2 cooperatively transactivate the distal ST2 promoter inducing expression of ST2, but not sST2 (36). Loss of PU.1 significantly decreased ST2 expression (36). Conversely, a PMA-responsive element has been found near the proximal promoter region of ST2 in the mouse fibroblast line NIH 3T3 (37). Similarly, activated human fibroblast line TM12, which only uses the proximal promoter for *Il1rl1* transcription, led to sST2 expression (32). These data further suggest that the distal promoter is used to transcribe ST2 and the proximal promoter is used to transcribe sST2. To verify these results and find other transcription factors important in ST2 and sST2 expressions, ChIP-seq experiments should be performed.

### ST2

ST2 was first found in serum-stimulated BALB/c-3T3 cells in the presence of cycloheximide (38). It contains an extracellular domain, which binds IL-33 with the help of IL-1 receptor accessory protein (IL-1RAP), a transmembrane domain, and an intercellular domain called a Toll/interleukin-1 receptor (TIR) domain. Due to the presence of the TIR domain, ST2 has been classified as a member of the IL-1 receptor superfamily. ST2 is expressed on cardiomyocytes (39) and a large variety of immune cells, including T conventional cells, particularly type 2 (40), regulatory T cells (Tregs) (41), innate helper 2 cells [innate lymphoid cell type 2 (ILC2)] (42), M2 polarized macrophages (43), mast cells (44), eosinophils (45), basophils (46), neutrophils (46), NK (47), and iNKT cells (47). Signaling through ST2 in immune cells induces type 2 and Treg immune responses, IgE production, and eosinophilia (5, 40–42, 48).

### sST2

sST2 protein lacks the transmembrane and cytoplasmic domains contained on ST2 and contains a unique nine amino acid *C*-terminal sequence (35). *In vitro*, sST2 production has been shown to be enhanced by pro-inflammatory cytokines (IL-1β and TNF-α) in human lung epithelial cells and cardiac myocytes. In humans, sST2 can be not only produced spontaneously by cells in the lung, kidney, heart, and small intestine (49) but also produced after activation with IL-33 in mast cells (50) or anti-CD3/anti-CD28 in both CD4 and CD8 conventional T cells (51). In a murine GVHD model, it has recently been shown that intestinal Th17 and Tc17 cells produced large amounts of sST2 following alloreactivity (51). This enhanced sST2 presence has been shown to inhibit the production of the type 2 cytokines IL-4 and IL-5 but not the type 1 cytokine IFN-γ (52).

## ST2/IL-33 Signaling

### The Membrane-Bound Form of ST2 Signals through MyD88/NF-κB

**Figure 2:**
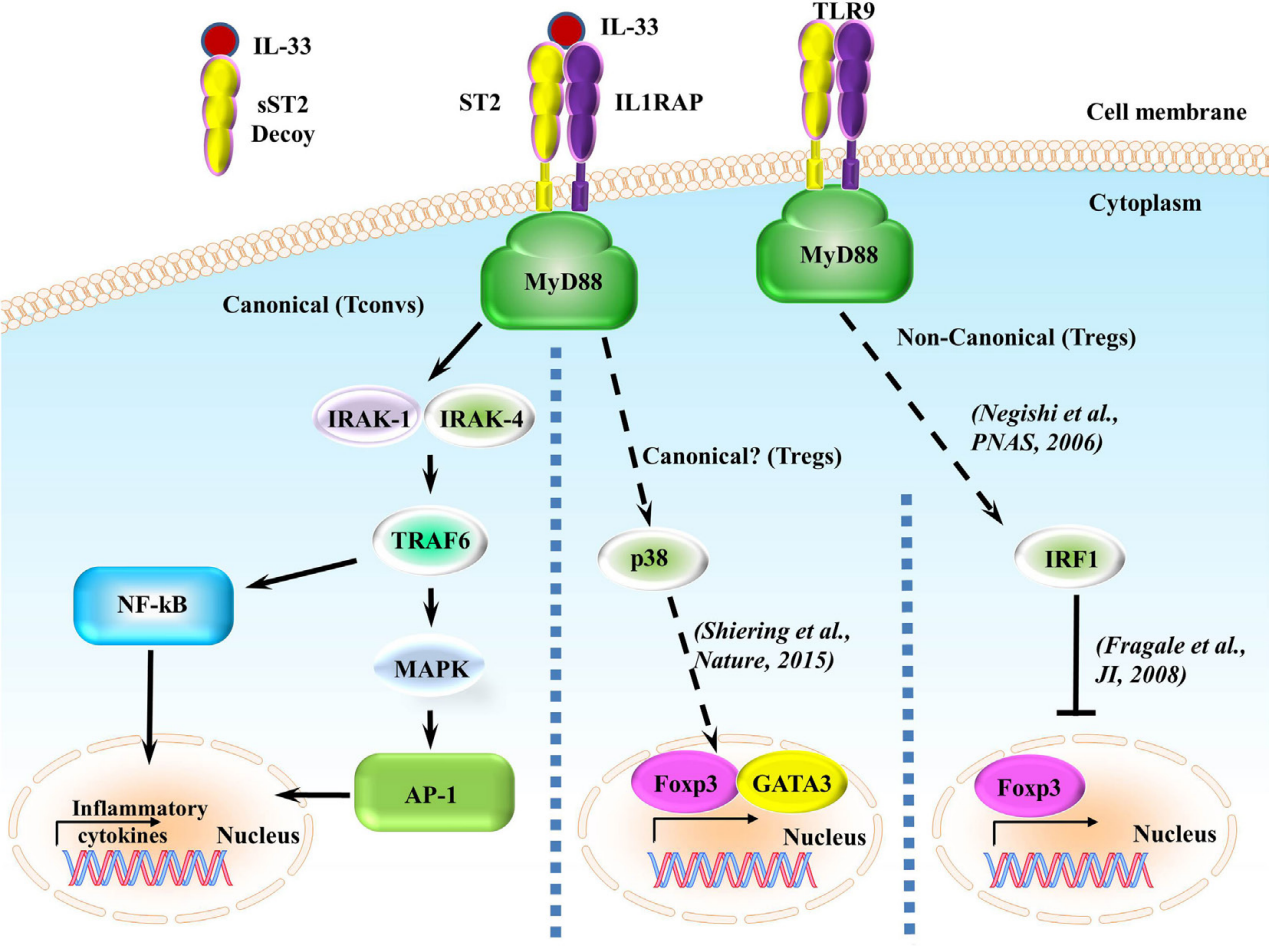
**ST2/IL-33 signaling pathway**. IL-33 either binds to the ST2/IL-1 receptor accessory protein (IL-1RAP) heterodimer, recruiting MyD88 to its intracellular domain, or the sST2 decoy receptor, which does not signal. MyD88 binding recruits IL-1R-associated kinase (IRAK) and TRAF6, leading to either the NF-κB or AP-1 pathway being activated. NF-κB and AP-1 activations promote inflammatory cytokine expressions. On regulatory T cells (Tregs), ST2/IL-33 signaling has been shown to promote Foxp3 and GATA3 expressions, while also promoting Treg function and expansion through enhancing TGF-β1-mediated differentiation though a p38-dependent mechanism. It has recently been shown that IFN regulatory factor (IRF) 1, which can be activated through MyD88 signaling, can inhibit Tregs by binding to the Foxp3 promoter and preventing Foxp3 transcription.

Upon IL-33 binding, the membrane-anchored ST2 forms a heterodimer along with IL-1RAP (53, 54) leading to the dimerization of the TIR domain. This leads to the recruitment of the TIR domain binding protein MyD88 and subsequent IL-1R-associated kinase activation, which can activate MAP kinases and NF-κB pathways (Figure 2) (5, 6). In regards to ST2/IL-33 signaling, how ST2/IL-33 signals specifically to either the MAPK or NF-κB is currently unclear. However, downstream events of ST2 do seem to occur differentially, as TRAF6 is required for NF-κB activation and induction of type 2 cytokines but TRAF6 is not needed for IL-33-induced ERK (a MAPK protein) activation (55). How TRAF6-independent activation of ERK occurs after IL-33 binding ST2 is currently unknown.

A recent report has shown that signaling through ST2/IL-33 in colonic Tregs helps to promote Foxp3 and GATA3 expressions while also promoting Treg function through enhancing TGF-β1-mediated differentiation (41). This enhancement is caused by phosphorylation of GATA3, which recruits more GATA3 and RNA polymerase II to the *Foxp3* promoter (41). GATA3 binds to the ST2 promoter, enhancing ST2 on the surface of both Th2 cells (56, 57) and Tregs (41, 57). IL-33 has been shown to drive NF-κB and p38 signaling in Tregs, leading to the selective expansion of ST2^+^ Tregs (58). As this effect is observed in Tregs in a non-diseased setting, independent of outside inflammatory responses, we believe that the ST2/IL-33-GATA3-Foxp3 pathway to be canonical. Conversely, in a non-canonical MyD88-dependent pathway (59), IFN regulatory factor (IRF) 1 signaling can inhibit Tregs by binding to the *Foxp3* promoter and preventing *Foxp3* transcription in murine T cells (60); however, this signaling leading to IRF1 activation through MyD88 has only been shown to be induced using CpG-B, a TLR9 agonist and a pathway independent from ST2/IL-33 (59). Whether ST2/IL-33 can activate IRF1 in a MyD88-dependent pathway and whether this ST2/IL-33-IRF1 activation can affect Treg function are currently unknown. We have highlighted the different ST2 signaling pathways in Figure 2.

Unlike IL-1RAP, the single immunoglobulin domain IL-1R-related molecule (SIGIRR or TIR8) SIGIRR can form a complex with ST2 upon IL-33 stimulation and can inhibit ST2/IL-33-mediated signaling both *in vitro* and *in vivo* (6, 61). IL-33 binding to ST2 has also been shown to negatively regulate ST2 through ST2 polyubiquitination, internalization, and degradation (62).

### The Soluble Form, (s)ST2, Is a Decoy Receptor and Does Not Signal

sST2 acts as a decoy receptor to sequester free IL-33, preventing ST2/IL-33 signaling. This was shown using a thymoma cell line transfected to express ST2, but not sST2, in the presence of added IL-33. When these thymoma cells were pretreated with sST2, they showed suppressed NF-κB activity (63). Another group used IL-33-treated cardiomyocytes and observed blocked prohypertrophic effects of angiotensin II or phenylephrines in the presence of sST2 (64). Blocking NF-κB signaling in lung alveolar epithelial cells and cardiac myocytes with the specific NF-κB inhibitor CAPE prevented sST2 production by these cells (49). In a human endotoxin model, healthy donors injected with LPS (2 ng/kg) had increased sST2 in their plasma within 24 h of injection (49). Fibroblast growth factor 2 enhanced sST2 production in the human breast adenocarcinoma cell line MCF-7 through MEK/ERK signaling (65). Lysophosphatidic acid has also been shown to increase sST2 production by human bronchial epithelial cells in an NF-κB- or JNK-dependent manner (66). Enhanced sST2 plasma circulation has been correlated with pulmonary fibrosis (67), acute myocardial infarction (39), subclinical brain injury and stroke (68), celiac disease (69), gastric cancer (70), HBV-related acute-on-chronic liver failure (71), HIV progression (72), and GVHD (17–24).

### IL-33 Regulation and Release

During cell stress or damage, IL-33 is released in either a full length or a cleaved form. Unlike IL-1β, however, IL-33 is not cleaved *via* caspase-1, and cleavage is not necessary for secretion nor biological activity of released IL-33, further suggesting its role as an alarmin (73, 74). Surprisingly, caspase-1, caspase-3, or caspase-7 processing actually leads to IL-33 inactivation (75, 76). Inactivation of IL-33 *via* caspases is therefore thought to alleviate the immune response, rather than enhance it. Other proteins are able to cleave IL-33, such as the neutrophil serine proteases cathepsin G and elastase, mast cell-derived serine proteases, tryptase, and chymase, and these proteins, unlike caspases, increase the biological activity of cleaved IL-33 10–30 times compared to that of full-length IL-33 (74, 77, 78).

IL-33 is expressed mainly by non-hematopoietic cells, including endothelial cells, adipocytes, fibroblasts, and intestinal and bronchial epithelial cells (8, 79, 80); however, some hematopoietic cells like dendritic cells (DCs) have also been show to express IL-33 when activated (5). In many non-hematopoietic tissues, IL-33 is constitutively expressed. Constitutive expression of IL-33 in epithelial cells suggests that IL-33 is used as an alarmin in response to infection or injury (8). Further suggesting IL-33 as an alarmin, IL-33 is released by damaged or necrotic cells (8), leading to activation of the immune system through ST2/IL-33 signaling (8, 81).

IL-33 can be found in the nucleus due to a nuclear localization sequence in the *N*-terminus, leading to binding of heterochromatin in the nucleus (7). Nuclear IL-33 can bind directly to NF-κB, sequestering it and preventing NF-κB signaling in HEK293RI cells, causing a downregulation of pro-inflammatory signaling (82). Further evidence of IL-33 having the ability to repress gene transcription is described because there is a structural similarity between a part of the IL-33 protein and the Kaposi sarcoma herpes virus motif latency-associated nuclear antigen (82). This mimicry allows IL-33 to bind to the H2A–H2B chromatin dimer and regulate the compaction of chromatin through nucleosome–nucleosome interactions. Recent discoveries have shown that nuclear IL-33 can bind to multiple sites in the promoter regions of ST2 in human endothelial cells and that knockdown of IL-33 increased sST2 levels (83). Loss of the nuclear localization domain of IL-33 led to non-resolving lethal inflammation (84). However, IL-33^−/−^ mice fail to develop autoimmune disease, and no one has shown whether nuclear IL-33 has been found in immune cells. These results indicate that nuclear IL-33 could act as a moderator of inflammation, but more evidence is needed to confirm the extent of the ability of nuclear IL-33 to moderate inflammation.

## ST2/IL-33 and Immune Cells

### ST2 Signaling on Lymphoid Cells

#### Th2 Cells

**Figure 3:**
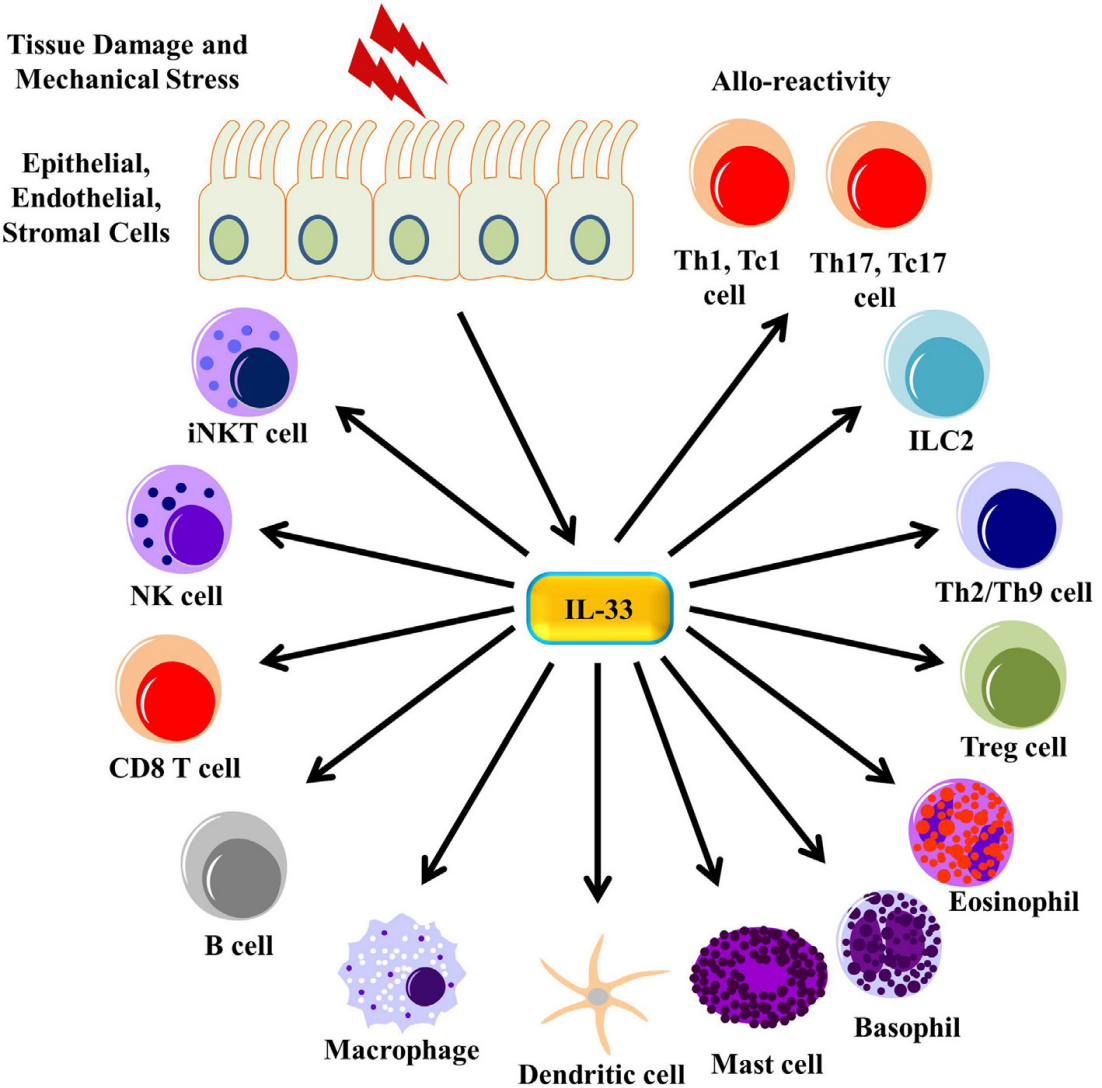
**IL-33 signaling on immune cells**. Tissue damage and mechanical stress to epithelial, endothelial, and stromal cells lead to the release of IL-33 from these cells. IL-33 then signals through many different immune cells, enhancing their function.

ST2 was first shown both *in vitro* and *ex vivo* to be preferentially expressed on murine Th2 cells (Figure 3; Table 1) expressing predominantly IL-4, IL-5, or IL-10, but not IFN-γ or IL-2 (40, 85). Its expression is independent of IL-4, IL-5, and IL-10, as loss of any of these cytokines does not affect ST2 expression on Th2 cells (40). ST2 expression on Th2 cells is dependent on GATA3 signaling (86) and is enhanced by IL-6, IL-1, TNF-α, and IL-5 (4). Given that ST2 expression in Th2 cells is independent of IL-4 and dependent on GATA3 signaling, it makes sense that ST2 expression occurs late during Th2 differentiation (4). IL-33 stimulation of Th2 cells *in vitro* increased the amount of IL-5 and IL-13 produced (5). Antigen-specific ST2^+^ Th2 cells were shown to produce more IL-5 and IL-13 compared to non-antigen-specific Th cells and ST2^−/−^ Th2 cells (87). Interestingly, IL-33 polarization of antigen-stimulated murine and human naïve CD4^+^ T cells leads to high IL-5 production but no IL-4 production, independent of GATA3 and STAT6 induction but dependent on MAPK and NF-κB signaling (88, 89). Adoptive transfer of these cells into naïve IL-4^−/−^ mice still triggered airway inflammation (88). *In vivo* administration of IL-33 led to an increase in the number of lymphocytes circulating in the blood and increased type 2 cytokine secretions in the thymus, spleen, liver, and lung (5). IL-33 has also been shown to be a chemoattractant for Th2 cells, as adoptive transfer of Th2 cells into ST2 knockout (KO) followed by IL-33 administration into the footpad of these mice led to the accumulation of the transferred Th2 cells (90). Loss of ST2 on Th2 during infection with the helminthic parasite *Nippostrongylus brasiliensis* did not affect Th2-mediated clearance of the infection nor was recruitment of Th2 cells in a murine model of asthma dependent on ST2, indicating that ST2 is not necessary for Th2 function (91). Recently, it was shown that human and murine Th2 cells do not produce sST2 *in vitro* (51).

**Table 1:** **ST2 and sST2 expressions and their regulation in immune cells**.

Cell type	ST2	ST2 regulation	sST2	sST2 regulation	Reference
Mast cell	+	Constitutively expressed	+	Strongly induced by IL-33 and weakly by Ag or SCF	(44, 50, 137–139)
Basophil	+	Induced by IL-3 stimulation	+	Released after IL-3 stimulation	(46, 145)
Eosinophil	+	Weakly constitutively expressed but strongly induced after IL-33 stimulation	?		(45)
Th2	+	Constitutively expressed and enhanced by IL-6, IL-1, TNF-α, and IL-5	−		(40, 51, 85)
Th1	−		+	Released after CD3 stimulation or alloactivation	(51)
Th17	−		+	Released after CD3 stimulation or alloactivation	(51)
Regulatory T cell (Treg)	+	Constitutively expressed only on Tregs expressing GATA3; enhanced by IL-33	−		(41, 58)
Th9	+	Constitutively expressed and enhanced by IL-33	?		(100, 101)
Innate lymphoid cell type 2	+	Constitutively expressed and enhanced by IL-33	−		(42, 109)
Dendritic cell	+	Weakly constitutively expressed but strong induction after rapamycin treatment	?		(134, 149)
Neutrophil	+	Weakly constitutively expressed	?		(46, 153)
Macrophage	+	Weakly constitutively expressed but enhanced by IL-4 and IL-13	+	Constitutively expressed	(43, 134)
B1 B cell	+	Constitutively expressed	?		(128)
iNKT cell	+	Constitutively expressed	?		(47, 130)
NK cell	+	Constitutively expressed	?		(47, 130)
Tc1 T cell	+	Weakly constitutively expressed	+	Released after CD3 stimulation or alloactivation	(51, 123, 124)
Tc17 T cell	−		+++	Released after CD3 stimulation or alloactivation	(51)

*+, expressed; −, not expressed; ?, unknown; Ag, antigen; SCF, stem cell factor; ST2, serum stimulation-2; sST2, soluble serum stimulation-2*.

#### Th9 Cells

IL-9-producing Th9 cells are the newest T cell subset to be described, polarized through TGF-β and IL-4 signaling (92, 93). When used separately on naïve T cells, TGF-β alone would cause Treg development, while IL-4 would induce Th2 cell differentiation. It has been found that the PU.1 gene is a Th9-specific transcription factor, which could induce IL-9 production in cells under Th2- or Th9-stimulating condition *in vitro* (94). Human or mouse PU.1-deficient T cells have diminished IL-9 production. Furthermore, IRF4 binds directly to the IL-9 promoter and is required for the development of Th9 cells, just like PU.1 (95). However, unlike PU.1, IRF4 is also required for the development of other Th cell subsets, including Th2 and Th17 cells (96, 97). Studies have shown that Th9 cells primarily secrete IL-9 to mediate the immune response in several diseases, such as asthma, autoimmune diseases, and parasitic infections (98), and IL-9 is associated with impaired Th1 immune response in patients with tuberculosis (99). Treatment of *in vitro* polarized human Th2 cells with TGF-β and IL-33 increases their IL-9 and ST2 expressions (100, 101).

#### Regulatory T Cells

ST2/IL-33 signaling in Tregs was first suggested to enhance their protective ability in an experimental colitis model in which IL-33 treatment ameliorated colonic tissue injury and colitis symptoms (41). IL-33 was shown to increase both ST2 and Foxp3 levels and expand Tregs in mice with colitis. ST2/IL-33 signaling in Tregs has also been shown to increase Treg frequency and decrease IL-17 and IFN-γ productions in an experimental autoimmune encephalomyelitis (EAE) model (102, 103). ST2^+^ Treg expansion is helped by IL-33 signaling in DCs, as IL-33 has been shown to stimulate DC production of IL-2, which selectively expands ST2^+^ Tregs (104). In a model of GVHD, treatment of mice daily with IL-33 from 10 days pretransplantation to day 4 posttransplantation enhanced the frequency of ST2^+^ Tregs, which persisted after irradiation, leading to disease amelioration through prevention of T conventional cell accumulation in target GVHD organs (58). Treatment of mice receiving a heart transplant with IL-33 has been shown to prolong graft survival through increase of Treg and myeloid suppressor-derived cell numbers (105, 106). Similarly, mice treated with IL-33 after skin transplantation had increased Treg numbers in the graft, decreased IFN-γ and IL-17 production, increased IL-10 production, and increased skin graft survival (107). This group also showed that ST2/IL-33 signaling can convert Foxp3^−^ CD4 cells into Foxp3^+^ CD4 Tregs in the periphery. We have shown that in a murine model of allogeneic hematopoietic stem cell transplantation (allo-HCT), transplanting ST2 KO Tregs with wild-type (WT) T conventional cells worsens GVHD compared to mice receiving WT T conventional cells and Tregs (51), further indicating the enhanced suppressive effect of ST2^+^ Tregs. Conversely to the enhanced protective effect of Tregs through ST2/IL-33, it has been reported that IRF1 is downstream of MyD88 (108) and negatively regulates *Foxp3* transcription (60, 108), although whether ST2/IL-33 signaling increases IRF1 expression, leading to decreased Treg function, has yet to be studied. Given that ST2/IL-33 has been shown to enhance, rather than impair Treg function, upregulation of IRF1 through MyD88 signaling is probably independent of ST2/IL-33. These data show that IL-33 signaling on Tregs increases their immunomodulatory function and could be further studied for their potential clinical benefits in a variety of diseases.

#### Innate Lymphoid Cells Type 2

Innate lymphoid cells type 2 were first discovered in the mouse and human mesenteries and found to be lineage marker negative, c-Kit positive, Sca-1 positive, IL-7Rα positive, and ST2 positive (42, 109). These cells have been shown to play a protective role against helminth infection and regulate metabolic homeostasis (110). In humans ST2^+^, ILC2s were later found in the lung and gut (111), and these ILC2s produced IL-5 and IL-13. During ILC2 activation, ST2 is upregulated in a GATA3- and Gfi1-dependent manner (112, 113). Treatment of Rag2 KO mice with IL-33 induced IL-5 and IL-13 production, whereas Rag2 and common gamma chain double KOs, which still have mast cells and basophils (both of which express ST2 and secrete type 2 cytokines), did not increase IL-5 or IL-13 production, indicating that this increase is due to ILC2 stimulation with IL-33 (42). ST2/IL-33 signaling enhancement was shown to expand ILC2s *in vivo* (42, 114). This group also found that ILC2s are major producers of type 2 cytokines after *Nippostrongylus brasiliensis* infection. It was also shown using the *N. brasiliensis* infection model that loss of both IL-33 and IL-25 signaling on ILC2s completely abrogated the early response against this infection due to impaired expansion of ILC2s and lack of IL-13 production, and adoptive transfer of WT ILC2s rescued this phenotype (42). During lung inflammation, ILC2s produce IL-9 (115), and IL-33 can promote cytokine production by ILC2s (116). Recently, it was shown that in a murine eosinophilic airway inflammation model that T-bet regulates IL-9 production by IL-33-stimulated ILC2s (117). ST2/IL-33 signaling in ILC2s is also important for protection against lung infection, as blocking ST2 signaling during influenza infection in mice lowered ILC2 frequency and number in the lung and resulted in diminished lung function, loss of airway epithelial integrity, and impaired respiratory tissue remodeling (118). Histological examination of influenza-infected lungs from anti-ST2-treated mice showed severe damage similar to that seen in a similar experiment where ILC2s were depleted (118). ILC2s have been recently reported to home to the skin in humans, where activation induces upregulation of ST2 (116). ST2/IL-33 signaling of ILC2s in the murine skin has been shown to not only promote atopic dermatitis (AD)-like inflammation (116, 119) but also promote skin wound repair (120). However, overstimulation of ILC2s with IL-33 during tissue remodeling of the liver after chemical injury promoted liver fibrosis (121). Also, signaling through ST2/IL-33 on ILC2s during breast cancer has been shown to promote breast cancer growth and metastasis (122). These data indicate that beneficial or harmful ST2/IL-33 stimulation in ILC2s is dependent on certain disease states.

#### CD8 T Cells

CD8 T cells have been shown to either express ST2 or produce sST2 (51, 123, 124). Although CD8 T cells express low levels of ST2, loss of either IL-33 or ST2 impaired the CD8 T cell response to LCMV infection (124). ST2/IL-33 signaling has also been shown to enhance CD8 T cell antitumor activity (125). During GVHD, however, IL-33 treatment during peak inflammation significantly increased GVHD severity and mortality in part through increased expansion of Tc1 cells (126). Given that IL-33 can increase type 1 responses when IL-12 levels are high (127), IL-33 treatment during peak inflammation was deleterious in this case.

#### B Cells

ST2 has been shown to be expressed on B1 B cells but not B2 B cells, leading to enhanced proliferation capacity and IgM, IL-5, and IL-13 productions both *in vitro* and *in vivo*; neutralizing IL-5 almost completely abolished this effect (128). Recent studies have also shown that IL-33 treatment in mice increases circulating IL-10-producing B cells that are neither conventional B1 nor B2 B cells (129). Adoptive transfer of these IL-33-treated, IL-10-producing B cells prevented spontaneous colitis in IL-10^−/−^ mice without affecting Treg frequency (129).

#### iNKT Cells and NK Cells

ST2/IL-33 signaling in murine iNKT cells causes their expansion and activation (130). Mice treated with IL-33 had twice as many iNKT cells in the spleen and liver compared to untreated mice (130). Unexpectedly, ST2 signaling in iNKT cells induced IFN-γ instead of IL-4 upon TCR engagement, which synergized in the presence of IL-12 (47, 130). This effect was also seen in Vα24^+^ human iNKT cells (47). NK cells constitutively express ST2, and ST2/IL-33 signaling increases IFN-γ levels synergistically with IL-12 (47, 130). Loss of ST2 in Ly49H^+^ NK cells did not affect their development but did impair their ability to expand and protect against MCMV (131). These data have yet to translate to human disease.

### sST2 Expression in Lymphoid Cells

#### Th1 and Th17 Cells

Although much of the research on ST2/IL-33 signaling in T conventional cells has been devoted to type 2 signaling, recent studies have come out looking at ST2/IL-33 signaling in type 1- and type 17-mediated diseases. Blockade of IL-33 with 200 µg anti-IL-33 every other day from day 0 until day 18 post-MOG_35–55_ injection during MOG-induced EAE ameliorated the disease in part through decreased IL-17 and IFN-γ productions, and treatment of 50 µg/kg IL-33 during this same time course enhanced IL-17 and IFN-γ productions (102). However, the amount of IL-33 given here is not physiological, so caution must be advised when interpreting these data. Conversely, another group using the same EAE model found that treatment with 1 µg IL-33 daily from day 12 to day 20 after immunization reduced IL-17 and IFN-γ productions and alleviated the disease (103). Seemingly, timing of ST2/IL-33 signaling affects response, perhaps through differing environments. In a murine model of collagen-induced arthritis, treatment with anti-ST2 antibody reduced both IFN-γ and IL-17 productions (132). In a murine model of rheumatoid arthritis, treatment with an sST2-Fc fusion protein attenuated disease and decreased production of IFN-γ, TNF-α, and IL-6 (133). Recently, we were the first to show that both murine and human Th1 and Th17 cells produce sST2 *in vitro* and *in vivo* after allo-HCT (51). Blocking ST2 with a blocking antibody *in vivo* decreased sST2 production in intestinal T cells 10 days after allo-HCT while maintaining ST2. Recipients of ST2^−/−^ T cells, compared to WT T cells, showed lower frequencies of T cells expressing the Th1 transcription factor T-bet and the Th17 transcription factor RORγt and their associated cytokines IFN-γ and IL-17, respectively, while increasing the expressions of the Th2 transcription factor GATA3 and the Treg transcription factor Foxp3 and their associated cytokines IL-4 and IL-10, respectively (51). Importantly, anti-ST2 treatment did not lead to loss of immunomodulatory ST2^+^ Tregs but rather maintained them in the intestine. On the basis of our findings, we have suggested that increased sST2 production affects the normal balance of pathogenic Th1/Th17 cells and immunomodulatory Th2/Treg cells by promoting the Th1/Th17 response and dampening the ST2-mediated Th2/Treg response through sequestering IL-33 (51).

#### Tc1 and Tc17 Cells

We were also the first to demonstrate that CD8 T cells, particularly Tc1 and Tc17 cells but not Tc2 cells, produce significant amounts of sST2 *in vitro* and after allo-HCT due to alloreactivity (51). sST2 secretion by donor T cells significantly increased as GVHD progressed. Similar to CD4 T cells, blocking ST2 with a blocking antibody decreased sST2 production by Tc1 and Tc17 cells *in vivo* after allo-HCT (51). Our data indicate that sST2 secretion by Tc1 and Tc17 cells sequester free IL-33, preventing ST2/IL-33-mediated Th2/Treg responses. In patients with early HIV infection, sST2 levels were strongly correlated with CD8 T cell count and their expressions of the activation markers HLA-DR and CD38 (72). However, it is not known if sST2 was produced from the CD8 T cells themselves or if sST2 is only a marker of gut damage and disease progression. While our study was the first to show that preventing sST2 secretion from CD8 T cells prevented disease pathogenesis, further studies are warranted to determine their role in other disease pathogeneses.

### Myeloid Cells

#### Macrophages

Macrophages, mast cells, basophils, eosinophils, and DCs all have been shown to express ST2 (43–46, 134). IL-33 amplifies the expression of M2 markers on murine macrophages (43, 135). Bone-derived human macrophages have been shown to constitutively express both ST2 and sST2; however, skewing these macrophages toward an M2 phenotype using IL-4 and IL-13 increased the expression of ST2 while not affecting sST2 expression (136). ST2/IL-33 signaling has been shown to enhance the activation of macrophages by upregulating the LPS receptor components TLR4 and MD2, soluble CD14, and MyD88 (135).

#### Mast Cells

ST2/IL-33 signaling on both murine and human mast cells has been shown to promote their survival through upregulation of B-cell lymphoma-X large in the peritoneum (137). ST2/IL-33 signaling also promotes mast cell activation and maturation, as IL-33 treatment of CD34^+^ mast cell precursors accelerated their maturation *in vitro* and induced GM-CSF, IL-5, IL-13, CXCL8, CCL17, CCL22, and CCL2 secretions (138, 139). These cytokine and chemokine secretions may be NFAT and AP-1 signaling dependent (140). It is well documented that mast cells can produce a variety of type 2 cytokines after ST2 signaling (141–143); however, ST2/IL-33 signaling on mast cells during airway inflammation has also been shown to promote a Th17 response (144).

#### Basophils and Eosinophils

ST2/IL-33 signaling promotes not only type 2 cytokine secretions such as IL-4 and IL-13 but also IL-8 in synergy with IL-3 or Fcε receptor activation on basophils (145). Basophils can also release sST2 after activation *via* IL-3 and C5a or anti-FcεRIα antibody, while IL-33 prevents sST2 release (145). IL-33 induces the degranulation of eosinophils and production of superoxide (45); controls their responsiveness to Siglec 8 (146); and increases IL-13, TGF-β, CCL3, CCL17, and CCL24 in the lungs during airway inflammation (147). Treatment with anti-ST2 antibodies prevented the upregulation of CD11b and decreased survival of eosinophils (148).

#### Dendritic Cells

Dendritic cells express low basal levels of ST2 on their cell surfaces (134); however, activation of DCs with rapamycin strongly upregulates ST2 through autocrine IL-1β signaling (149). Treatment of DCs with IL-33 has been shown to increase surface levels of MHC-II, CD40, CD80, CD86, OX40L, and CCR7 (134, 150, 151). ST2/IL-33 signaling in DCs also increases their productions of IL-4, IL-5, IL-13, CCL17, TNF-α, and IL-1β (150). In the presence of naïve CD4^+^ T cells, IL-33-activated DCs induce IL-5 and IL-13 but not IL-4 and IFN-γ from the T cells (134, 151). Interestingly, sST2 has also been shown to be internalized by DCs, suggesting a non-canonical method of action for sST2 (152). It is currently unknown whether sST2 can be internalized by other immune cells. IL-33-activated murine DCs have recently been shown to be required for *in vitro* and *in vivo* expansion of ST2^+^ Tregs through DC IL-2 production (104), which could be used for therapeutic benefit against inflammatory diseases through expansion of Tregs both *in vitro* and *in vivo*. ST2 expression on host hematopoietic cells, including DCs, and non-hematopoietic cells was not implicated in the severity of GVHD as recipient ST2 KO bone marrow chimeras did not modify GVHD severity (51).

#### Neutrophils

While ST2 has been shown to be present on neutrophils (46, 153), not much is known about the role of ST2 on neutrophils. It has been shown that IL-33-treated murine and human neutrophils do not downregulate CXCR2 induced by the activation of TLR4 through the inhibition of GRK2 (153). IL-33 injected into the ears of mice induced neutrophil recruitment to the skin (154); however, it is not clear if ST2/IL-33 signaling on the neutrophils directly led to their migration.

## ST2/IL-33 in Inflammatory Diseases

### Gastrointestinal Diseases

#### Inflammatory Bowel Disease

It is believed that IBD starts with a dysregulated immune response to either food or commensal gut bacteria, leading to the production of pro-inflammatory cytokines such as TNF-α, IL-6, IL-1, and IL-8. Expression of these cytokines along with chemokine release leads to attraction of T cells, specifically type 1 T cells, to the intestines. Continual damage of the gut mucosa by these type 1 cells and other immune cells such as macrophages, neutrophils, and DCs leads to the release of various alarmins and other proteins. sST2 was found to be significantly increased in both the gut mucosa and the serum in both patients and experimental models of IBD (13–16). However, in IBD patients, ST2 expression remained similar to that of healthy patients (13). In the lamina propria of active ulcerative colitis (UC) patients, ST2 predominately came from CD11b^+^ and CD4^+^ cells (14). These findings suggest that increased sST2 production by lymphocytes or the gut mucosa could either lead to development of IBD, particularly UC, or that these proteins are markers for disease severity.

ST2/IL-33 signaling has been shown to enhance epithelial proliferation and mucus production in the gut (5), suggesting that the increase in IL-33 in the colonic mucosa in active UC could be beneficial. However, in multiple mouse models of IBD, use of ST2 KO mice led to amelioration of IBD compared to WT controls. These results were verified using an IL-33 KO. Using bone marrow chimeras, it was shown that ST2 signaling in non-hematopoietic cells was responsible for IBD. This was due to ST2/IL-33 signaling impairing epithelial barrier function and delayed wound healing. Lack of ST2 signaling in hematopoietic cells did not prevent UC development. A ST2 blocking antibody confirmed the findings from the KO experiments (155). Crohn’s disease (CD), however, shows opposite results from UC. In a trinitrobenzene sulfonic acid-induced IBD model, which mimics the pathology of human CD, administration of recombinant IL-33 (rIL-33) into mice ameliorated colonic tissue injury and clinical symptoms (156). Protection was shown to be through upregulation of type 2 cytokines, Foxp3^+^ Tregs, and CD103 DCs, which promote Treg development. In patient colons with active IBD, Treg levels in the lamina propria are increased compared to healthy controls and function normally (157, 158). It has recently been shown that colonic Tregs preferentially express ST2 and that signaling through ST2/IL-33 promotes both Treg accumulation and maintenance in the intestine and enhances their protective function (41). However, treatment with rIL-33 to promote Treg-mediated protection may be time dependent, as rIL-33 treatment at onset of a DSS-induced colitis model exacerbated disease severity. rIL-33 treatment during recovery or chronic phases ameliorated DSS-induced colitis (159). Given these data, selective treatment of ST2^+^ Tregs with IL-33 could provide therapeutic benefits.

#### Graft-versus-Host Disease

**Figure 4:**
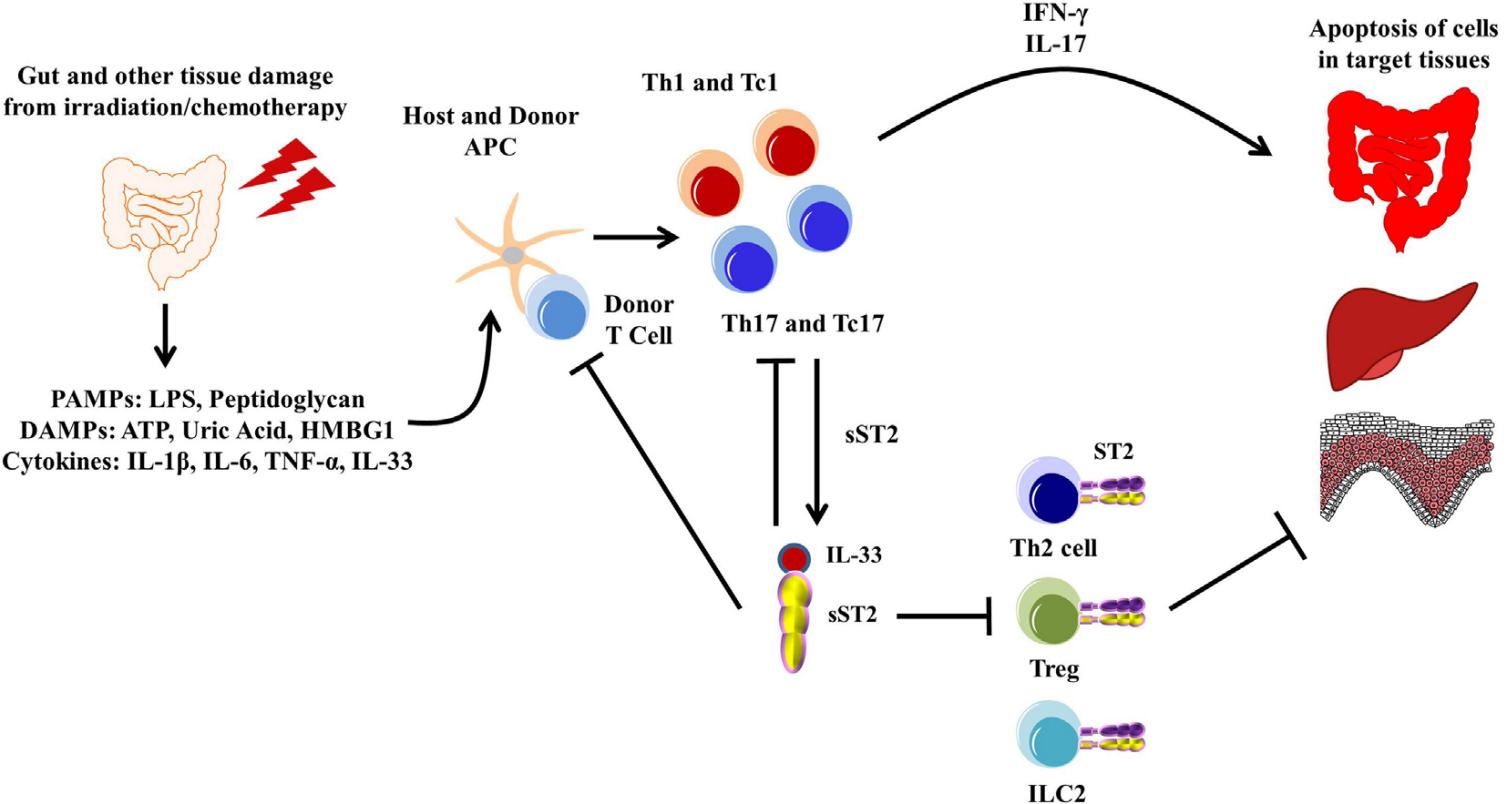
**Pathogenesis of graft-versus-host disease (GVHD)**. The gut and other issues are damaged during irradiation or chemotherapy, leading to the release of various DAMPs, PAMPs, and cytokines, including IL-33. These DAMPs, PAMPs, and cytokines activate both host and donor antigen-presenting cells (APCs), which then activate the donor T cells. The APCs are also secreting various cytokines that promotes T cell differentiation toward type 1 and type 17 responses. These activated type 1 and type 17 T cells are able to secrete various pro-inflammatory cytokines, leading to apoptosis of healthy tissue, mainly in the gut, liver, and skin, which can be exacerbated by free IL-33. Furthermore, sST2 is produced by both type 1 and type 17 T cells, and while this may sequester free IL-33 from the type 1 and type 17 T cells, sST2 can also prevent the potential beneficial effects from ST2/IL-33 signaling in Th2 cells, Tregs, and lymphoid cell type 2 (ILC2s).

Graft-versus-host disease is a common occurrence in patients who undergo allo-HCT as treatment for both malignant and non-malignant diseases of the blood and bone marrow. The pathogenesis of GVHD has been well documented and is now thought to occur in three steps: (1) activation of antigen-presenting cells caused by tissue damage from the conditioning regimen leading to the release of pro-inflammatory cytokines and danger signals, (2) alloactivation of donor T cells leading to their proliferation and differentiation into type 1 and type 17 T cells, and (3) tissue destruction by alloreactive T cells through release of cytolytic molecules leading to donor cell apoptosis, mainly in the mucosal tissues (160). Discovering prognostic and diagnostic biomarkers for GVHD has been successful with sST2 being one of the most validated to date (17–24). Blocking sST2 with a blocking antibody during the peritransplant period decreased GVHD morbidity and mortality in both minor histocompatibility and humanized murine models (Figure 4). Importantly, the ST2-blocking antibody, which inhibits the full length of ST2 and not specifically sST2, maintained protective ST2-expressing T cells while also not impairing the graft-versus-leukemia activity (51), suggesting that addition of anti-ST2 or a ST2 small molecule inhibitor could show efficacy in reducing GVHD-related morbidity and mortality in patients. Using IL-33 as a treatment seems to be time dependent, as injections with IL-33 during the peak inflammatory response in a murine model led to increased morbidity and mortality in mice due to increased migration and increased pro-inflammatory cytokine production (126). IL-33 treatment preconditioning, however, increased the number of ST2^+^ Tregs, which persisted after irradiation in a murine model. This led to decreased GVHD severity and mortality. Adoptive transfer of ST2^+^ versus ST2^−^ Tregs showed that GVHD protection is increased by ST2^+^ and not ST2^−^ Tregs (58). Given that IL-33 is pleiotropic, IL-33 treatment for GVHD seems to be dependent on both timing and the state of inflammation present.

#### Other Gut Diseases

ST2/IL-33 signaling has been implicated in protection from various infections, which could impact the gut. Studies have shown that treatment of mice with rIL-33 led to epithelial cell hyperplasia in the gut along with infiltration of eosinophils and mononuclear cells in the lamina propria (42, 161). These effects are thought to be mediated by IL-13, which becomes overexpressed after IL-33 treatment (5). Treatment of mice with IL-33 after *Trichuris muris* infection increased parasite clearance through increased Th2 cytokine response (161). Other infections that can impact the gut, including *Toxoplasma gondii* (162), *Leptospira* (163), and *Pseudomonas aeruginosa* (164), have shown that the loss of ST2 or high sST2 levels led to higher morbidity and mortality with increased Th1 cytokine profiles. Recent studies have shown that gut epithelial barrier dysfunction and immune activation independently predict mortality during treated HIV infection (165). A later study showed that patients during the early stage of HIV infection, defined as being within 180 days of the date of infection, had higher levels of sST2 in their plasma and was highly correlated with CD8 T cell count and levels of gut mucosal damage, but not with viral load or CD4 T cell count (72).

sST2 increase has also been implicated during small bowel transplant rejection (25). Patients who had rejection of small bowel transplants had higher serum levels of sST2 during rejection compared to that during rejection-free time points and that rejection increased allograft ST2 expression. An increase in sST2 in the allograft was predicted by Pathway and Network Analysis caused by TNF-α and IL-1β signaling (25). However, these data do not implicate sST2 as a mediator of disease but rather a biomarker of occurring transplant rejection.

### Lung Diseases

#### Asthma and Allergy

Asthma is characterized by varying levels of airway hyperresponsiveness, mucus secretion, bronchoconstriction, and chronic inflammation, affecting 300 million people worldwide (166). ST2/IL-33 signaling in mast cells, ILC2s, eosinophils, basophils, Th2, and Th9 cells drive allergic asthma (141–143, 147, 167–170). Activating ST2 signaling in bone marrow-derived mast cells and basophils *in vitro* shows strong type 2 cytokine production, including IL-4 and IL-13 (171). Treatment of mice with IL-33 alone induced airway hyperresponsiveness and goblet cell hyperplasia through IL-4, IL-5, and IL-13 induction in the lungs (172). RAG2^−/−^ mice treated with IL-33 also induce this phenotype, implicating the importance of the innate immune system in generating airway inflammation (171). IL-33 has also been shown to be a chemoattractant for Th2 cells, as injection of IL-33 into the footpad of ST2^−/−^ mice, which were adoptively transferred with WT polarized Th2 cells led to local accumulation of the transferred Th2 cells (90). After ovalbumin (OVA)-induced acute allergic lung inflammation, levels of IL-33, ST2, and sST2 are significantly increased in the lung (172). While activation of the innate immune system in part through ST2/IL-33 signaling establishes airway inflammation, ST2^+^ T cells maintain this inflammation. Indeed, injecting mice with a ST2 blocking antibody during the resolution phase after OVA-induced allergic inflammation, when Th2 cells but not eosinophils are present in the lung, reduced airway hyperresponsiveness and mucus production (173). Loss of both IL-4 and IL-5 during OVA-induced airway inflammation did not abolish airway hyperreactivity, which was abolished only after anti-CD4 treatment (174), suggesting that ST2/IL-33 signaling in CD4 T cells may be critical for efficient antigen-induced airway inflammation (175). Blockade of either IL-33 or addition of sST2 before OVA-induced allergic airway inflammation showed reduced total cell counts and eosinophil counts in the bronchoalveolar lavage fluid and decreased IL-4, IL-5, and IL-13 (40, 52, 176).

#### Non-Allergic Lung Disease

During idiopathic pulmonary fibrosis, patients with stable disease versus healthy controls had similar levels of serum sST2; however, during exacerbations of fibrosis, there was over a sixfold increase in serum sST2 in the exacerbation group compared to both healthy controls and stable disease groups, which correlated with measurements of inflammation (67). Patients with acute respiratory distress syndrome had significantly higher sST2 levels at day 0, which were associated with worse prognosis and mortality (177). Serum levels of sST2 were also increased in patients with chronic obstructive pulmonary disease compared to control patients (178). Patients with moderate or severe H1N1 influenza infection also had significantly increased serum sST2 compared to patients with mild H1N1 infection or rhinovirus infection (179). Idiopathic pneumonia syndrome (IPS) occurs in 5–10% of patients who undergo allo-HCT. In a cohort of 42 patients with IPS without infection, sST2 was significantly higher in the serum of patients with IPS compared to healthy controls and patients with human rhinovirus and parainfluenza occurring at approximately the same time after transplantation (180).

### Skin Diseases

#### Atopic Dermatitis

Atopic dermatitis is a Th2-driven disease and patients with AD show higher IgE levels and eosinophilia in the skin and blood (181). ST2/IL-33 signaling has been recently implicated in AD, as transgenic mice expressing IL-33 under the keratin 14 promoter had spontaneous AD-like inflammation (119). This led to increased IL-5 producing ILC2s. Mice receiving topically applied OVA, house dust mites, or staphylococcal enterotoxin B led to upregulation of both sST2 and IL-33 expressions (182). Patients with AD have higher IL-33 expression levels in their skin lesions compared to healthy controls (182). However, in a small cohort of 71 adults and 61 children with AD, serum sST2 levels were not significantly elevated compared to adult controls, in contrast to sST2 expression in patient skin lesions (182, 183). Currently, phase I–II clinical trials are being conducted using novel anti-IL-33 antibodies.

#### Psoriasis and Vitiligo

Unlike AD, psoriasis is driven by Th1 and Th17 cytokines (184). However, psoriatic skin still shows increased IL-33 expression compared to healthy skin in patients (154). ST2^−/−^ mice had reduced cutaneous inflammatory responses compared to WT mice in a phorbol ester-induced murine model of psoriasis (154).

Patients with vitiligo are characterized by the disappearance of their melanocytes. In lesions of patients with vitiligo, both ST2 and IL-33 levels were increased, and serum levels of IL-33 were increased (185). As ST2 signaling in psoriasis and vitiligo is relatively new, not much else has been published as of yet.

#### Scleroderma and Chronic GVHD

Scleroderma is characterized by the fibrosis and hardening of the skin and connective tissues, measured by the modified Rodnan skin score (MRSS) test (186). In a two cohort study, serum sST2 levels were increased in patients with scleroderma compared to healthy controls, which, when combined with Spondin-1, best described longitudinal change in MRSS, using mixed linear models (187). This was validated using three other independent cohorts (187).

Chronic GVHD can affect multiple organs, with skin involvement being one of the most common. In a large study of chronic GVHD, plasma samples were collected from patients at day +100 post-allo-HCT. A four-biomarker panel, which included sST2, correlated strongly with chronic GVHD diagnosis, severity, and non-relapse mortality (21).

### Cardiac Diseases

sST2 was found to be upregulated after mechanically stimulating cardiomyocytes and stimulating with IL-1β (39). Inducing myocardial infarction *via* coronary artery ligation increased sST2 in the serum of mice compared to unoperated controls (39). This observation was also seen in patients, as those who suffered myocardial infarction had elevated serum sST2 levels 1 day postevent (39). In a cohort of over 800 patients with acute ST-elevation myocardial infarction, sST2 levels 1 day postevent correlated with 30-day mortality independent of age, blood pressure, heart rate, infarct territory, and time from symptom onset to treatment (11). sST2 levels of patients with non-ischemic congestive heart failure at time of entry to the study also correlated with both brain natriuretic peptide levels (BNP), which is routinely used in the clinic and serum noradrenaline levels (12). This study also found that changes in serum sST2 were an independent predictor of mortality. sST2 levels are correlated with impaired epicardial coronary flow and risk of death or congestive heart failure within 30 days of presentation, independent of BNP (188). These data show the value of sST2 as a biomarker in cardiac diseases.

Functional analysis of ST2/IL-33 signaling and sST2 production has shown that treatment of cultured rat neonatal cardiomyocytes with rIL-33 blocked angiotensin II or phenylephrine induced hypertrophy, while addition of sST2 or blocking of ST2 with an antibody reversed this effect (64). When using ST2^−/−^ or WT mice to look at *in vivo* response to pressure overload by transverse aortic constriction, ST2^−/−^ mice had more left ventricular (LV) hypertrophy, more chamber dilation, reduced fractional shortening, more fibrosis, and impaired survival compared with WT mice (64). Treatment of WT mice with rIL-33 reduced fibrosis and hypertrophy and increased survival in WT mice (64). This reduction in damage when treating with IL-33 may be due to inhibited apoptosis in cardiomyocytes (189).

In an atherosclerosis model in which mice deficient for the ApoE protein fed a high-fat diet, treatment with rIL-33 reduced aortic atherosclerotic plaque development and increased levels of type 2 cytokines in the serum (190), which have an atheroprotective effect. Mice treated with sST2 developed significantly larger atherosclerotic plaques (190). These data indicate that ST2/IL-33 signaling may have a protective effect, while sST2 plays a deleterious role in cardiac diseases.

### Obesity and Metabolic Complications

Accumulation of visceral adipose tissue (VAT) due to obesity leads to inflammation, insulin resistance, and development of type 2 diabetes (191), leading to the reduction and function of Tregs in the VAT (192), which have been shown to be enriched for ST2 expression (193, 194). IL-33 is critical for the development and maintenance of these VAT Tregs (194). *In vitro* culturing of murine adipocytes with IL-33 induced IL-5 and IL-13 production, decreased expression of genes associated with adipogenesis and lipid metabolism, and reduced lipid storage (195). ST2^−/−^ mice fed a high-fat diet had increased body weight and fat mass and impaired glucose regulation and insulin secretion compared to high-fat diet WT controls (195). IL-33 treatment to genetically obese diabetic mice led to reduced adiposity, lower fasting glucose levels, improved glucose and insulin tolerance, accumulation of Th2 cells and M2 macrophages in their adipose tissue, and increased the proportion of ST2^+^ Tregs in the VAT (193, 195). However, ST2/IL-33 signaling may only help obesity-related insulin resistance. An age-associated insulin resistance model showed that fat-resident Treg depletion protected against insulin resistance, and these findings were confirmed using an anti-ST2 antibody (196).

ST2 and IL-33 are produced by white adipose tissue and in preadipocyte and adipocyte cell cultures in humans (79), while sST2 expression has been shown to be increased in omental and subcutaneous human adipose tissues (197). In a large, population-based study, sST2 levels in the plasma of patients strongly correlated with markers of diabetes, after adjusting for age and gender (28). In another study separating 525 patients into normal, prediabetic, and diabetic groups, plasma sST2 levels were only significantly increased in the diabetic group compared to prediabetic and normal groups (29). In a multicenter, cross-sectional study of 180 patients with metabolic syndrome with normal LV ejection fraction, LV mass index was independently associated with serum sST2 concentrations. Increased sST2 associated with increased likelihood of LV hypertrophy and increased systolic blood pressure (198). New-onset posttransplantation diabetes mellitus (PTDM) is a common occurrence after allo-HCT. Serum sST2 levels from three cohorts collected at engraftment and day 30 showed elevated sST2 levels at both time points and that high sST2 levels predicted PTDM and non-relapse mortality, independent of conditioning and high-grade GVHD (27). These data suggest that high sST2 levels correlate with obesity and type 2 diabetes and metabolic complications even when sST2 is already elevated by alloreactivity.

## Potential Therapeutic Benefit of Targeting ST2/IL-33 Signaling

The clinical usefulness of targeting either sST2 excess of secretion or ST2/IL-33 excess signaling or use of sST2 as a biomarker for diseases has been a hot topic in the last few years, as shown by the increase in translational studies devoted to ST2/IL-33 and sST2. Manipulation of ST2/IL-33 signaling or blocking sST2 secretion or sequestration of IL-33 is highly disease dependent. Several new antibodies that inhibit IL-33 binding to ST2 are currently being tested in phase I–II clinical trials for patients with asthma and chronic obstructive pulmonary disease. Using either an antibody or small molecule inhibitor is an attractive option for therapeutics targeting sST2 in CD, GVHD, or heart disease, while ideally maintaining ST2. However, given the involvement of ST2/IL-33 in a multitude of processes, caution must be afforded.

sST2 usefulness as a clinical biomarker has been studied extensively in both cardiac and allo-HCT patients, showing both prognostic and diagnostic value (11, 12, 17–24, 27, 39, 187, 188). sST2 levels are also increased in patients suffering from intestinal (13–16) and metabolic diseases (27, 28, 197, 198); however, the data from these studies so far are correlative and have not passed the qualification for biomarkers that can be used in clinic (199).

## Conclusion

ST2/IL-33 signaling in immune cells has recently become a hot target of study. This signaling helps to activate T cells, ILC2s, DCs, B cells, mast cells, basophils, eosinophils, and other immune cells. Most of the work has shown that ST2/IL-33 signaling enhances the type 2 response, although recent studies have shown how ST2/IL-33 signaling enhances the immunomodulatory effects of Tregs. T cells have also been recently shown to produce sST2, which was once thought to be produced only by non-hematopoietic cells. ST2/IL-33 signaling in Tregs, ILC2s, and IL-10-producing B cells protects against inflammation, while sST2 can act either as a biomarker or can play a role in a variety of diseases by sequestering IL-33 and preventing ST2/IL-33 signaling. However, ST2/IL-33 signaling can also lead to progression of various lung and skin diseases such as asthma and AD. Given the complexity between ST2/IL-33 signaling and timing during the immune response and the importance of ST2/IL-33 in various organ systems, several questions and challenges remain. When does ST2/IL-33 signaling affect Treg response more so than the inflammatory response in various diseases? Which mediators can enhance ST2 expression on immunomodulatory cells? Which mediators can reduce or promote sST2 production during disease? A better understanding of the impact of ST2/IL-33 and sST2 during disease and how ST2 and sST2 targeting could affect different organ systems will be critical for the development of therapeutics.

## Author Contributions

Both BG and SP devised, wrote, and revised the manuscript.

## Conflict of Interest Statement

SP has a patent on “Methods of detection of graft-versus-host disease” licensed to Viracor-IBT Laboratories. Otherwise, the other author has no other relevant conflicts of interest to declare.
